# Use of a self-rating scale to monitor depression severity in recurrent GP consultations in primary care – does it really make a difference? A randomised controlled study

**DOI:** 10.1186/s12875-016-0578-9

**Published:** 2017-01-19

**Authors:** C. Wikberg, J. Westman, E-L. Petersson, M. E. H. Larsson, M. André, R. Eggertsen, J. Thorn, H. Ågren, C. Björkelund

**Affiliations:** 1 Department of Primary Health Care/Public Health and Community Medicine, Institute of Medicine, Sahlgrenska Academy, University of Gothenburg, Gothenburg, Sweden; 2 Division for Family Medicine, Department of Neurobiology, Care Sciences and Society, Karolinska Institutet, Stockholm, Sweden; 3 Region Västra Götaland, Närhälsan Research and Development Primary Health Care, Gothenburg, Sweden; 4 Department of Health and Rehabilitation, Unit of Physiotherapy, Institute of Neuroscience and Physiology, University of Gothenburg, Gothenburg, Sweden; 5 Department of Public Health and Caring Sciences - Family Medicine and Preventive Medicine, Uppsala University, Uppsala, Sweden; 6 Institute of Neuroscience and Physiology, University of Gothenburg, Gothenburg, Sweden

**Keywords:** Depression, Primary care, Self-assessment instrument, Adherence, Sick-leave, Quality-of- life

## Abstract

**Background:**

Little information is available about whether the use of self-assessment instruments in primary care affects depression course and outcome. The purpose was to evaluate whether using a depression self-rating scale in recurrent person-centred GP consultations affected depression severity, quality of life, medication use, and sick leave frequency.

**Methods:**

Patients in the intervention group met their GP regularly at least 4 times during the 3 months intervention. In addition to treatment as usual (TAU), patients completed a self-assessment instrument (Montgomery-Asberg Depression Rating Scale) on each occasion, and then GPs used the completed instrument as the basis for a person-centred discussion of changes in depression symptoms. The control group received TAU. Frequency of visits in the TAU arm was the result of the GPs’ and patients’ joint assessments of care need in each case.

Depression severity was measured with Beck Depression Inventory-II (BDI-II), quality of life with EQ-5D, and psychological well-being with the General Health Questionnaire-12 (GHQ-12). Data on sick leave, antidepressant and sedatives use, and care contacts were collected from electronic patient records. All variables were measured at baseline and 3, 6, and 12 months. Mean intra-individual changes were compared between the intervention and TAU group.

**Results:**

There were no significant differences between the intervention and control group in depression severity reduction or remission rate, change in quality of life, psychological well-being, sedative prescriptions, or sick leave during the whole 12-month follow-up. However, significantly more patients in the intervention group continued antidepressants until the 6 month follow-up (86/125 vs 78/133, *p* < 0.05).

**Conclusions:**

When GPs used a depression self-rating scale in recurrent consultations, patients more often continued antidepressant medication according to guidelines, compared to TAU patients. However, reduction of depressive symptoms, remission rate, quality of life, psychological well-being, sedative use, sick leave, and health care use 4-12 months was not significantly different from the TAU group. These findings suggest that frequent use of depression rating scales in person-centred primary care consultations has no further additional effect on patients’ depression or well-being, sick leave, or health care use.

**Trial registration:**

ClinicalTrials.gov Identifier: NCT01402206. Registered June 27 2011(retrospectively registered).

**Electronic supplementary material:**

The online version of this article (doi:10.1186/s12875-016-0578-9) contains supplementary material, which is available to authorized users.

## Background

Depression is a leading cause of disability and affects 10 to 15% of the population of most countries in the world [1, 2]. A majority of patients with depression are treated in primary care, and approximately 75% of antidepressants are prescribed by general practitioners (GPs) [3, 4]. The usual treatment for depression in Swedish primary care, as in primary care in other countries, depends upon symptom severity. Most people treated for depression in primary care receive one or more of the following: early reassessment (watchful waiting) and symptom monitoring by their GP, cognitive behavioural therapy or other psychotherapy, and antidepressant medication [5, 6].

Depression guidelines recommend that GPs regularly use self-rating scales to evaluate and monitor symptoms in patients with depression [5, 6]. These guidelines, however, have been based largely on clinical expertise rather than on the results of randomised controlled trials [3]. Furthermore, most self-rating scales recommended for detecting and monitoring depression were designed in psychiatric care settings and have not been adapted for use in primary care [3]. Little information is thus available about whether the use of such instruments in primary care affects depression symptoms, treatment, rehabilitation, or recovery [7]. We know that using such scales for screening does not improve detection or management of depression in settings such as primary care [8]. However, we do not know whether using a self-rating scale to follow the course of depression in person-centred primary care consultations improves treatment and outcomes of depression.

The depression rating scale most commonly used in Swedish primary health care is the Montgomery-Asberg Depression Rating Scale [9]. However, its use has mainly been evaluated in psychiatric settings [9, 10]. MADRS-S is sensitive to changes in symptoms of depression and is therefore a valuable tool in person-centred consultations [10–12]. However, a qualitative study based on interviews with Swedish GPs showed that many GPs thought that MADRS-S was of limited utility in consultations [13]. It is thus still unclear whether or not future guidelines should recommend mandatory use of depression self-rating scales in primary health care.

The aim of the current study was to evaluate in a randomised control trial whether using a depression rating scale (MADRS-S) in recurrent person-centred GP consultations affected depression severity, quality of life, overall psychological well-being, antidepressant and sedative use, sick leave, and health care use in a long time perspective.

## Methods

The PRI-SMA study (PRImary care Self-assessment Montgomery-Asberg) was a multicentre, controlled trial that took place at primary health care centres (PHCCs) and was randomised at the GP level. The GPs randomised to the intervention used a patient self-rating scale at recurrent monthly patient visits in addition to providing usual care. The GPs randomised to the control condition provided treatment as usual (TAU). The intervention lasted for 3 months. Adult patients who presented with a recent depressive episode (mild/moderate depression) at the PHCC were recruited to the study by the GPs.

The trial took place at 22 Swedish PHCCs between March 2010 and December 2013. All 98 PHCCs in the region were invited to participate in the intervention; 22 agreed to participate.

### Randomisation of GPs at primary health care centres

All GPs took part in an information meeting about the study. All GPs also met with the study leaders when the leaders visited each participating PHCC at the time of the intervention start-up at that PHCC. Before the intervention started, the GPs at each PHCC were randomised to either intervention treatment or TAU. All GP names were written on slips of paper and mixed in a container, and an administrative employee blinded to the aim of the trial drew names. The GP whose name was first drawn was assigned to the intervention group, the GP whose name was drawn second was assigned to the control group, and so on until all names were drawn.

### Study procedure

Patient recruitment took place at one PHCC at a time. During the 2 week recruitment period, a research nurse worked full-time at the PHCC to facilitate patient recruitment, collect patient data, and support PHCC staff.

### Inclusion of patients

Study participants were patients aged 18 and up who visited the PHCCs and were identified and diagnosed by a GP with a new episode of mild/moderate depressive disorder [14].

During the 2 week recruitment period, all randomised GPs who met patients they suspected of having depression used the PRIME-MD diagnostic instrument to confirm or reject the diagnosis of depression in accordance with DSM-IV criteria [14]. All patients who fulfilled the diagnostic criteria for depressive disorder, i.e. mild to moderate (BDI score ≤36), were asked if they would like to participate in the study.

Inclusion criteria were: written informed consent, age ≥18 years, diagnosed with mild to moderate depressive disorder and either not prescribed antidepressant medication or had no changes in antidepressant medication during the preceding 2 months.

Exclusion criteria were: lack of written informed consent, antidepressant medication introduced or changed during the 2 months prior to baseline, diagnosed with severe depressive disorder (BDI-II >36, confirmed by diagnostic procedure by GP), diagnosed with severe mental disorder (i.e., bipolar disorder, antisocial personality disorder, psychosis, substance use disorder, or other serious mental disorder), suicidal ideation or earlier suicide attempts, did not speak or understand Swedish, and/or had cognitive disabilities that made it difficult or impossible to complete the assessment instruments, including MADRS-S.

### Intervention

The intervention consisted of using a patient depression self-rating scale (MADRS-S) in recurrent monthly consultations during the 3-month intervention. Patients made 4 visits to their GPs, at which time they completed MADRS-S to monitor changes in their depressive symptoms that were then discussed in the person-centred consultation [15, 16]. MADRS-S was used as a supplement to, rather than as a substitute for, TAU.

The GPs randomised to the group that provided the intervention received four hours of guidance about how to include the results of MADRS-S in the person-centred consultation. The intervention GPs also received a video CD with the same pre-recorded information. The person-centred consultations involved patients and GPs collaborating to increase patients’ ability to manage their depression. The guidance therefore included a reminder to the GPs that MADRS-S was used for the sake of the patients rather than the GPs.

### Treatment as usual

The GPs randomised to the group that would provide TAU were instructed to manage patients with depression the same way they usually did (but with the addition of the diagnostic procedure in the initial consultation). In general Swedish GPs are very knowledgeable about and use of person-centred consultation methods in their daily practice of the kind described in Maguire 2002 [16].

Three months after baseline, patients in the control group were followed up in an appointment with a nurse at the PHCC.

### Data collection procedure

A study nurse collected data from participants in the intervention and control groups during the first visit (baseline), at a follow-up visit to the PHCC at the end of the intervention (3 months after baseline), and by postal questionnaires 6 and 12 months after baseline (see Additional files 1 and 2).

### Measures

#### Background variables

Background information was obtained by a questionnaire at the patients’ first visit to the PHCC. Age was measured as age in years, gender as female or male. Marital status was dichotomised as single or married/cohabiting. Children living at home was dichotomised as participants who had children <18 years living at home vs. participants not having children living at home. Educational level was identified as as lower educational level (primary school or vocational school), middle educational level (high school) and higher educational level (college or university). Employment status was dichotomised as working/studying vs. unemployed/retired.

Place of birth was dichotomised as born in one of the Nordic countries vs. born outside the Nordic countries. Smoking status was categorised as “non-smokers” (participants who had not smoked during the last year), “sometimes smokers” (those who only smoked a single cigarette per week or more seldom), and “smokers” (more frequent smoking). Leisure-time physical activity was categorised as i) no or almost no physical activity during leisure time, ii) activity ≥4 hrs. per week, and iii) intensive (several times a week and vigorously).

#### Outcomes

Depression severity; Assessed before (at baseline) and after the intervention with the Beck Depression Inventory-II (BDI-II) and categorised as mild (BDI-II score >13-19)*,* moderate (BDI-II score 20-28), and high moderate (BDI-II score, 29-36) [17, 18].

Remission; Identified as a BDI-II score ≤ 13 at 3-month follow up.

Quality of life; Measured with the standardised EQ-5D instrument [19, 20].

Overall psychological well-being; Assessed with the 12-item General Health Questionnaire (GHQ-12) (response scale 0-3) [21].

Prescriptions for antidepressants; Information on this measure was obtained from patients’ electronic patient records (EPRs) and from responses to the patient questionnaires and dichotomised as yes (used) or no (did not use). Guidelines indicate that treatment should not be stopped before 6-12 months after recovery [5].

Prescriptions for sedatives; Information on prescriptions for sedatives was obtained from patients’ electronic patient records (EPRs) and from responses to the patient questionnaires and dichotomised as yes (used) or no (did not use).

Sick leave; Information on sick leave was obtained from EPRs and responses to the patient questionnaires. Sick leave was used both as a dichotomous variable (yes or no) and as number of days of sick leave.

Health care use; This measure comprised information on number of visits to GPs, number of visits to nurses, number of visits to psychologists/psychotherapists, and total number of visits to the PHCC. Information was obtained from EPRs by study personnel and divided into the number of visits between 0 and 3 months, and 4 and 12 months.

### Statistical methods

The student’s t-test (paired) was used to compare mean intra-individual changes in the intervention and TAU groups. Two-sided Chi-square or Mann Whitney U-tests were used to compare frequencies. Statistical significance was set at p < 0.05. Logistic regression models were used to examine whether changes in the BDI-II, EQ-5D, and GHQ-12 variables from baseline to 3, 6 and 12 months follow-ups were associated with sex, age, education, antidepressant medication, and group assignment (intervention/TAU).The results are presented as odds ratios (OR) with 95% confidence intervals (CI). All analyses were performed in SPSS. All analyses were conducted on an intention to treat basis.

### Power calculation

Power was calculated for the BDI-II-score outcome on the basis of empirical experience of the variation in this factor, taking into account that GPs were the unit of randomisation. The power estimate was based on the assumption of an alpha error = 0.05 and a beta error = 0.20 (power = 0.80). Given an expected effect difference of 2 units (BDI-II) over time between the intervention and TAU groups, we calculated that we would need 105 patients in each group to achieve sufficient statistical power. To safeguard for dropouts, we recruited 20% more patients than the power calculation indicated that we needed.

### Ethics

The Regional Ethical Review Board in Gothenburg, Sweden approved this study (Dnr 746-09; T612-10). Prior to inclusion and after receiving oral and written information about the study, participants provided written informed consent. The trial was registered at ClinicalTrials.gov (Identifier: NCT01402206) [22].

## Results

**Fig. 1:**
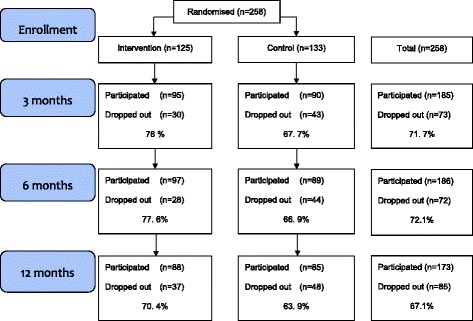
PRI-SMA-Study (CONSORT Flowchart)

Of the 91 participating GPs, 45 were randomised into a group that provided the intervention and 46 into a group that provided TAU. The participating GPs enrolled 258 patients, and the flow of the patients through the study is shown in Fig. 1 [23]. There was no significant difference in participation rate in the patients in the intervention and control groups at the 3-, 6- or 12-months follow-ups.

Table 1 presents the background characteristics of the study population. No significant differences were found between the participants in the intervention and TAU groups at baseline.

**Table 1:** Baseline characteristics of participants in the PRI-SMA trial (*n* = 258)

Characteristics	Total *n* (%)	Intervention *n* (%)	TAU^¤^ *n* (%)	*p*
Participants	258	125	133	
Age (years, mean )	43.48	44.84	42.19	0.2
men	76 (29.5)	31 (24.8)	45 (33.8)	
women	182 (70.5)	94 (75.2)	88 (66.2)	0.1
Marital status				
single	118 (45.7)	61 (48.8)	57 (42.9)	
married/cohabiting	140 (54.3)	64 (51.2)	76 (57.1)	
children <18 years at home	82 (43.2)	36 (40.4)	46 (45.5)	0.5
Lower educational level	34 (13.2)	17 (13.7)	17 (12.8)	
Middle educational level	115 (44.7)	61 (49.2)	54 (40.6)	
High educational level	108 (42.0)	46 (37.1)	62 (46.6)	0.3
Employment				
working/studying	181 (80.8)	90 (81.1)	91 (80.5)	
unemployed/retired	43 (19.2)	21 (18.9)	22 (19.5)	0.9
Born outside the Nordic countries	41 (16)	19 (15.3)	22 (16.7)	0.8
Smoking (yes or sometimes)	75 (29.4)	36 (29.3)	39 (29.5)	0.9
Leisure-time physical activity				
never	109 (42.4)	57 (46)	52 (39.1)	
at least 4 hrs/week	124 (48.2)	55 (44.4)	69 (51.9)	
intensive	24 (9.3)	12 (9.7)	12 (9,0)	0.5
Depression				
mild (BDI-II 12-19)	32 (13)	16 (13.6)	16 (12.4)	
moderate (BDI-II 20-28)	82 (33.2)	39 (33.1)	43 (33.3)	
high moderate (BDI-II 29-36)	68 (27.5)	35 (29.7)	33 (25.6)	0.9

^¤^ *TAU* treatment as usual

There was a statistically significant difference between participants and drop-outs during the study concerning age (mean age 44.3 in participants, mean age 37.3 in drop-outs, *p* = 0.02), gender (male 14/62, 22.6%, female 16/166, 9.6%, *p* = 0.034), and ethnicity (born in Sweden 21/194, 10.8%, and born outside Sweden 9/32, 28.1%, *p* = 0.035)

**Fig. 2:**
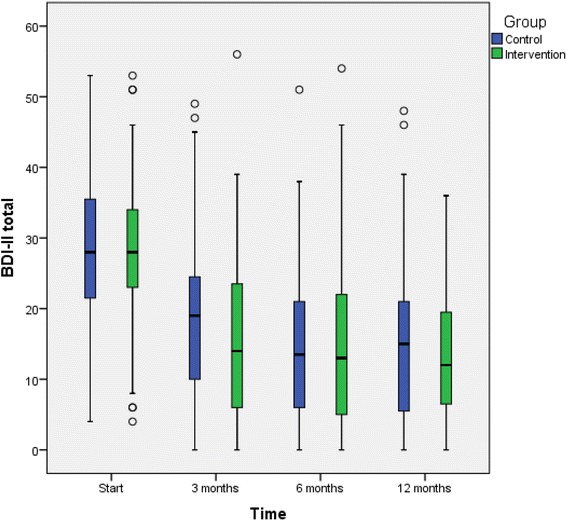
Depression severity (BDI-II values) in the intervention and TAU groups. Boxplots for baseline, 3-months follow up, 6-months follow up, and 12-months follow up. The outcome variable is presented as box plot with medians, minimum and maximum values and lower and upper quartiles. Y-axis: BDI-II, X-axis: time 0, 3, 6, 12 months. Outliers (*circle*) are cases with values between 1.5 and 3 times the interquartile range (5 cases with BDI-II ≥50 in patients with difficulties in Swedish language, where complementary diagnostic procedure by the GP ended up on medium depressive disorder)

**Fig. 3:**
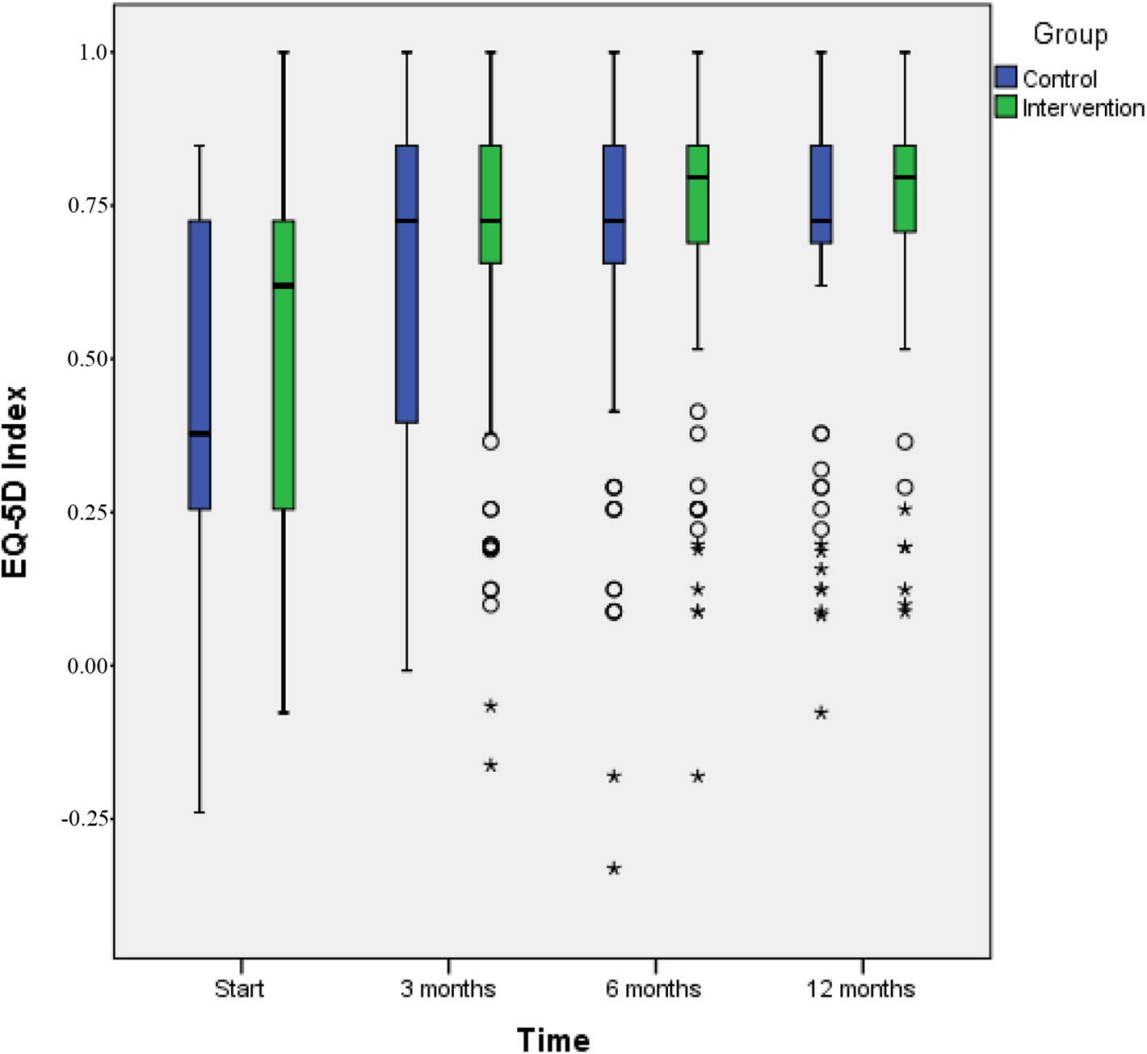
Quality of life (EQ-5D values) in the intervention and TAU groups. Boxplots for baseline, 3-months follow up, 6-months follow up, and 12-months follow up. The outcome variable is presented as box plot with medians, minimum and maximum values and lower and upper quartiles. Y-axis: EQ-5D, X-axis: time 0, 3, 6, 12 months. Outliers (*circle*) are cases with values between 1.5 and 3 times the interquartile range, extremes (*star*) are cases with values more than 3 times the interquartile range

**Fig. 4:**
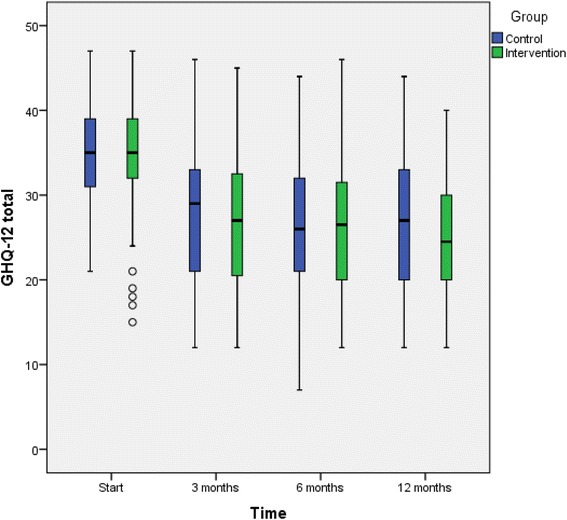
Overall psychological well-being (GHQ-12 values) in the intervention and TAU groups. Boxplots for baseline, 3-months follow up, 6-months follow up, and 12-months follow up. The outcome variable is presented as box plot with medians, minimum and maximum values and lower and upper quartiles. Y-axis: GHQ-12, X-axis: time 0, 3, 6, 12 months. Outliers (*circle*) are cases with values between 1.5 and 3 times the interquartile range

Figures 2, 3, and 4 shows depression symptoms (BDI-II), quality of life (EQ-5D), and overall psychological well-being (GHQ-12) in intervention and TAU groups at baseline and 3, 6, and 12 months (boxplots). All patients who participated in the study, both those in the intervention and those in the TAU group, improved in all three variables between baseline and 3 months but there were no significant differences in mean changes (mean of intra-individual ∆) between the groups. Improvements were substantial in both groups; at the 3-month follow-up, nearly half the patients no longer had depression (BDI <13) (49% in the intervention and 47.3% in the TAU group, *p* = 0.89). We also performed logistic regression analyses. The models showed that at the 12-month follow-up, older age was associated with improved BDI-II score (OR 1.05, CI 1.01-1.99), and a higher level of education was associated with improved EQ-5D score (OR 3.9, CI 1.77-8.73). The factors added to the model (sex, age, education, antidepressant medication, and participation in the intervention or TAU group) explained the same amount of variation in both the intervention and TAU groups. Thus, the difference between the groups remained non-significant (data not shown). No adverse events were reported from intervention or TAU group.

Table 2 shows differences between the intervention and TAU groups in the percent of patients who had prescriptions for antidepressants and sedatives. At baseline, 22% of patients in the intervention group and 32% of patients in the TAU group were on maintenance antidepressant medication. After 3 months, the proportion of patients with prescriptions for antidepressant medication was 72% in both the intervention and the TAU group. At the 6-month follow-up, the proportion of patients taking antidepressants had dropped to 69% in the intervention group and 59% in the TAU group (p = 0.007). At the 12-month follow-up, there were no significant differences in the percent of patients who had prescriptions for antidepressants or sedatives in the intervention and TAU groups (Table 2).

**Table 2:** Number and percent of patients who had prescriptions for antidepressants and sedatives and stated medication at baseline and 3-, 6-, and 12-months follow up, respectively, in the intervention and TAU groups

	Baseline		3 months follow-up		6 months follow-up		12 months follow-up	
	*n*	(%)	*n*	(%)	*n*	(% )	*n*	(%)
Antidepressants								
Intervention (n = 125)	27	(22)	90	(72)	86	(69)**	74	(59)
TAU (n = 133)	43	(32)	96	(72)	78	(59)	77	(58)
Sedatives								
Intervention	26	(21)	64	(51)	53	(42)	52	(42)
TAU^¤^	32	(24)	73	(55)	56	(42)	51	(38)

^¤^ *TAU* treatment as usual

**Significantly higher proportion of patients in intervention group still on antidepressants after 6 months, *p* = 0.007

Table 3 shows the mean number of days of sick leave by patient group. There were no significant differences in the percentage of patients on sick leave in the intervention and TAU groups between baseline and 3 months, or 4 -12 months, or during the entire study period. The mean total duration of sick leave (days) was not significantly different between the intervention and TAU groups (Table 3).

**Table 3:** Number and percent of individuals on sick leave, mean days of sick leave, and p-values for difference in mean days of sick leave between participants in intervention and TAU groups, based on information obtained from electronic patient records

	Intervention				TAU		
	*n* (%)	Mean days of sick leave	SD	*n* (%)	Mean days of sick leave	SD	*P* for difference mean days
0-3 months	31(25)	63.1	29.8	48 (36)	55.8	27.7	0.221
4-12 months	35(28)	100.8	87.3	47(35)	102.7	85	0.922
Total 0-12 months	49(39)	124.8	102.5	64 (48)	123.3	85.0	0.942

### Health care use

Mean number of visits at the PHCC, including visits to GP, nurse, psychologist/therapist and total visits to PHCC, was compared between intervention and TAU groups. All information was obtained from EPRs (Table 4). We also compared the total number of visits. There were no significant differences between the intervention and TAU groups regarding these outcomes, during the total 12-month follow-up period. However, during the 0-3 month period, the TAU group made significantly more visits to psychologists/psychotherapists than the intervention group and the intervention group made significantly more total visits to the PHCC than the TAU group.

**Table 4:** Number of patients’ contacts with GPs, nurses, and psychologists between baseline to 3 months, and 4 to12 months for intervention and TAU groups, based on information obtained from electronic patient records

Profession	Type of contact	Intervention 0-3 months m (SD)	TAU 0-3 months m (SD)	*p*
GP	visit	3.44 (1.214)	2.59 (1.354)	0.066
Nurse	visit	0.32 (0.829)	0.32 (0.875)	0.878
Psychologist/Therapist	visit	0.40 (1.320)	0.89 (1.776)	**0. 0001**
Total visits to PHCC	visit	4.16 (2.398)	3.81 (2.692)	**0.006**
		Intervention 4-12 months m (SD)	TAU 4-12 months m (SD)	*p*
GP	visit	2. 54 (2.337)	2.38 (2.595)	0.301
Nurse	visit	0. 68 (1.484)	0.62 (1.551)	0.581
Psychologist/Therapist	visit	0. 90 (2.504)	0.83 (1.908)	0.401
Total visits to PHCC	visit	4. 11 (4.586)	3.83 (4.403)	0.619
		Intervention total 0-12 months m (SD)	TAU total 0-12 months m (SD)	*p*
GP	visit	8.26 (5.842)	7.64 (5.976)	0.304
Nurse	visit	0.99 (1.978)	0.94 (2.088)	0.967
Psychologist/Therapist	visit	1.30 (3.129)	1.73 (3.222)	0.167
Total visits to PHCC	visit	8.26 (5.842)	7.64 (5.976)	0.812

Bold numeral indicates statistically significant *p*-value

## Discussion

In this RCT, which evaluated use of a depression self-rating scale in recurrent person-centred GP consultations, more patients in the intervention than control group adhered to antidepressant treatment for the recommended 6 months. However, compared to TAU, there were no significant differences between the effects on depression symptom reduction, quality of life or overall psychological well-being, sedative use, sick leave, or health care use. These findings suggest that recurrent use of depression self-assessment scales in person-centred primary care consultations does have an effect on antidepressant medication adherence, but has no further additional effects, compared to the treatment usually provided in Swedish primary care.

### Strengths and weaknesses of the study

The intervention was conducted in ordinary primary care rather than advertising for participants, which would have resulted in a sample that was not representative of patients in primary health care. The study compared the intervention with a TAU group rather than using a control group that consisted of patients on a waiting list. Studies of the effects of cognitive behavioural therapy show that being on a waiting list has a nocebo effect (i.e. results in worsening depression symptoms), which in turn makes intervention effects appear larger than they really are [24, 25]. We had access to all patients’ EPRs, a good source of information on prescriptions, sick leave, and health care use. In the current study, we had the opportunity to observe the course of depression symptoms, perceived quality of life, sick leave, and health care use for a full year after baseline. Another major strength is that a research nurse was stationed at each PHCC during the recruitment period. The nurse collected baseline data, gathered oral and written consent, and supported the staff at the site, functions that primary care personnel stress are important to make it easier to combine clinical work and research [26].We were well aware of the possible obstacles to conducting a RCT at a PHCC as described by Richter and Roy-Byrne [27, 28].

We randomised at the GP level. Randomisation at the patient level would have necessitated changing doctor for some patients, or GPs trained in the intervention would have provided the intervention to some patients but treatment as usual to others, increasing the possibility of contamination. We chose not to randomise at the PHCC level, as regional and socioeconomic differences between areas served by the PHCCs might have affected the results.

Another limitation was absence of a blinded control arm. The control GPs were aware that their colleagues at the same PHCC were providing the intervention, and this might have affected treatment as usual, even if they were requested to continue patient care as usual. This possible bias would support the notion of randomisation at PHCC level, but this would in turn increase the risk of e.g. selection effects. Another limitation is that the intervention of recurrent use of self-rating scales also included regular GP visits, and it is not possible to separate possible effects of the regular GP visits from effects of the patient’s frequent self-rating monitoring. However, during the 3-month intervention, patients in the TAU arm made only 0.85 fewer visits to their GPs than patients in the intervention arm. The frequency of visits in the TAU arm was the result of the GPs’ and patients’ joint assessments of care needs in each case. That frequency of patient visits did not differ markedly in the TAU arm is likely attributable to the current wide-spread emphasis on person-centred care in Swedish primary care.

All patients included had a new episode of mild/moderate depression, but the selection procedure did not differentiate between recurrent or first episode patients. There is some evidence that recurrent depression has slower remission [29], but according to the similar frequency of maintenance antidepressant medication in both intervention and TAU groups at inclusion, we can assume that the rate of recurrent depression disorder was the same in both arms. Further, the high number of outcomes, assessed at baseline and at 3, 6, and 12 months, necessitated multiple statistical comparisons, thus increasing the possibility of type I errors. Although the low p-values indicated that the few significant findings were not likely due to chance, the results need further confirmation in other studies. A limitation is also that we did not use more advanced statistical models to account for the correlation between patients treated by the same GP and to handle all observations of the patient instead of pairwise comparisons (e.g. mixed models).

### Drop outs

Among the study drop outs, 37 individuals belonged to the intervention group and 48 to the TAU group. At the 3 and 6 month follow-up, participation rate was somewhat lower in the control group, but at the 12 month follow up, participation rate was almost the same in intervention and control groups; 70% and 69%, respectively (Fig. 1).

### Generalisability

The study population represented patients treated by GPs for mild and moderate depression in primary health care. It included patients from health care centres in urban and rural areas and areas characterised by differing socioeconomic status. Health care is heavily subsidised in Sweden; patients pay a small per-visit fee and their yearly total co-payment is capped at approximately £100. The study results can be generalised to other countries with similar health care systems.

The RCT had a representative selection among the Swedish speaking population. One could argue that the exclusion of non-speaking Swedish patients may have limited the study’s generalisability. Unfortunately, it was not possible to translate and validate the instruments or to use an interpreter at every contact with study staff.

In this study, we chose to use MADR-S as the basis for patient education and collaborative care. These two factors can facilitate better depression outcomes [29–31]. Depression is complex, and the outcome of treatment often depends on the GP’s ability to connect with the patient during the consultation and her/his ability to choose proper treatment in agreement with the patient. Use of self-rating scales may function as one piece of the depression treatment puzzle, but as in many difficult jigsaw puzzles, more than one piece is needed to complete the picture. Thus, the Swedish National Board of Health and Welfare, i.e. the body that establishes the national guidelines for depression treatment, has expressed interest in whether it is meaningful to routinely use self-rating scales for patients with mild to moderate depression in primary care. We chose to use MADRS-S because it is the most commonly used depression self-rating scale in Sweden. About one third of all GPs state that they sometimes use MADRS-S, although not regularly [13, 32]. MADRS-S is designed to be sensitive to change and is often used in RCT studies that evaluate antidepressant treatment [12].

The study shows that use of self-rating scales during regular visits facilitates adherence to pharmacological treatment according to guidelines, in that once medication with antidepressants has been initiated, this treatment should be continued for at least 6 months [5]. On the other hand, the study also shows that treatment as usual, as it is practiced in the Swedish primary care of today, in most cases not including the use of self-rating scales, is just as efficient concerning reduction of depressive symptoms, perceived quality of life and general health, as well as sick leave frequency and duration, which is supported by the results from several international studies [33].

### Strengths and weaknesses in relation to other studies

To our knowledge there have been few or no studies that have evaluated the effect of recurrent use of self-rating scales regarding depression in relation to the above described outcomes.

Use of antidepressants for the period mandated by current guidelines was associated with neither an improvement nor a worsening of patients’ symptoms during the one-year study period. However, using antidepressants for the length of time called for in guidelines has been shown to reduce risk of relapse [34].

### Clinical importance of the study

More discussion is needed as to how self-rating scales might be best implemented in primary care, how to manage depressed patients using these instruments, and how to integrate this into the consultation. The feasibility of meaningful and person-centred use of self-rating scales, continuity, and accessibility in the care of mild to moderate depression in the clinical setting is of great interest. Engaging the patient with a cognitive and person-centred approach and with regular consultations and follow-ups is a way to empower the patient, give the patient knowledge and tools to handle depression, follow his or her illness course, and create a basis for regaining of function [2, 6, 30]. Continuity and close regular follow-ups facilitate care that is adjusted to the individual's explicit needs and requirements and is an absolute condition for person-centred care [35]. Requirements for treatment are also reached in cooperation and collaboration with the patient, facilitating understanding and communication [36]. The continuous use of self-rating scales in the treatment of primary care patients with depression might support this process of care.

## Conclusions

When GPs used a depression self-rating scale in recurrent consultations, patients more often continued antidepressant medication according to guidelines, compared to TAU patients. However, concerning outcomes in terms of depression symptoms, quality of life, overall psychological well-being, consumption of care, and sick leave the use of self-rating scales did not seem to increase treatment effects compared to the usual treatment provided in primary care. Thus, the use of depression self-rating scales should perhaps not be mandatory in primary health care but rather left to the discretion of the GP and the patient.

## Additional files

Additional file 1: Patient questionnaire 1 PRISMA background. (PDF 143 kb)
Additional file 2: Patient questionnaire follow up PRISMA 2. (PDF 136 kb)

## Abbreviations

GP: General practitioner
MADRS-S: Montgomery-Asberg Depression Rating Scale (Self-administered)
PHCC: Primary health care centre
PRI-SMA: PRImary care Self-assessment Montgomery-Asberg
RCT: Randomised controlled trial
