# Supplementary material for: Use of a self-rating scale to monitor depression severity in recurrent GP consultations in primary care – does it really make a difference? A randomised controlled study
Source: BMC Fam Pract. 2017 Jan 19;18:6. doi: 10.1186/s12875-016-0578-9 (PMC5244530; doi:10.1186/s12875-016-0578-9)
Supplement: Additional file 1: — Patient questionnaire 1 PRISMA background. (PDF 143 kb) [file 12875_2016_578_MOESM1_ESM.pdf]

GÖTEBORGS UNIVERSITET

VÄSTRA GÖTALANDSREGIONEN

Projekt MADRS-S/PRI-SMA in Primary Care **Date** ☐

Patient background questionnaire 1
Name

☐

Pers nb (10 digits) **ID nr:** gender nr
(given by the nurse)

☐ ☐☐☐☐☐☐

Age ……….

Are you
- Man ☐
- Woman ☐

Civilstånd
- Unmarried ☐
- Married ☐
- Partners ☐
- Divorced ☐
- Widow/widower ☐

Är du
- Living toghether ☐
- Living alone ☐

Number of children living at home under 18? ☐

Do you smoke?
- Yes ☐
- No ☐
- Sometimes ☐
- Have smoked but not last year ☐

Your education

Primary school	☐
Upper secondary	☐
University	☐
Special vocational	☐

What is your main occupation today?
Obs! Only one alternative.

1 ☐ Employee
2 ☐ Self-employed
3 ☐ Studying
4 ☐ On long timer sick-leave or early retirement
5 ☐ Childcare leave
6 ☐ In search for work
7 ☐ Working in my houshold
8 ☐ Pensioner

Profession ....

In which country were you born? ……………………………………

If you were not born in Sweden, when did you move here? Year ………………………

How often are you physycally active (running, bicycling, training etc) at least 30 minutes at one time?

1 ☐ 4 times or more per week
2 ☐ 2-3 times per week
3 ☐ 1 time per week
4 ☐ Some time per month
5 ☐ Never

How often are you physically active at leisure time?
If your activity changes much from summer to winter, try to make an average
The question concerns the latest year**.**

☐ **Mostly sitting in leisure time - sedentary**
Mostly reading, television, cinema, computer.

☐ **Medium physically active at leisure time**
Walking, bicykling, ore move in some way at least 4 hours per week.

☐ **Regular athletics or training**
Running, swimming, tennis, badminton, or hard work in the garden etc at least 2-3 hours a week.

☐ **Hard training or competitive sport**
Hard training several times a week

Are you on sick-leave just now?

No ☐

Yes ☐ Reason……………………………………………………………

If you are on sick-leave just now, when do you think you will be back at work part-or fulltime?

1 ☐ In 1- 4 weeks
2 ☐ In 2- 6 months
3 ☐ In 7 - 12 months
4 ☐ After 1 år
5 ☐ Never
6 ☐ Don't know

Do you use medicines regularly?

No ☐

yes ☐

I use the following medicines regularly:

……………………………………………………………………………………

……………………………………………………………………………………

……………………………………………………………………………………

Thanks for your cooperation!
