# Supplementary material for: Use of a self-rating scale to monitor depression severity in recurrent GP consultations in primary care – does it really make a difference? A randomised controlled study
Source: BMC Fam Pract. 2017 Jan 19;18:6. doi: 10.1186/s12875-016-0578-9 (PMC5244530; doi:10.1186/s12875-016-0578-9)
Supplement: Additional file 2: — Patient questionnaire follow up PRISMA 2. (PDF 136 kb) [file 12875_2016_578_MOESM2_ESM.pdf]

GÖTEBORGS UNIVERSITET

VÄSTRA GÖTALANDSREGIONEN

Project MADRS-S/PRI-SMA **Date** [ ]

Patient questionnaire 2 – follow-up

Name

[ ]

Pers nb (10 nb) **ID nr:** gender nr

(given by the nurse)

[ ] [ ][ ][ ][ ][ ][ ]

Age……….

What is your occupation now?

Obs! only one alternative.

1 ☐ Work as employee

2 ☐ Own employer

3 ☐ Studying

4 ☐ Long time sick-leave or early retirement

5 ☐ Parental leave

6 ☐ In search of work

7 ☐ Working at home with the household

8 ☐ Pensioner

Your occupation ....

Do you smoke?

Yes ☐

No ☐

Sometimes ☐

How often are you physically active (running, bicycling, training etc) at least 30 minutes at one time?

1 ☐ 4 times or more per week
2 ☐ 2-3 times per week
3 ☐ 1 time per week
4 ☐ Some time per month
5 ☐ Never

How often are you physically active at leisure time?
If your activity changes much from summer to winter, try to make an average
The question concerns the latest year. **Only tic one box.**

☐ **Mostly sitting in leisure time -sedentary**
Mostly reading, television, cinema, computer.

☐ **Medium physically active at leisure time**
Walking, bicykling, ore move in some way at least 4 hours per week.

☐ **Regular atheltics or trainig**
Running, swimming, tennis, badminton, or hard work in the garden etc at least 2-3 hours a week.

☐ **Hard training or competitive sport .**
Hard training several times a week

Have you been on sick-leave during the last 3 months?

No ☐

Yes ☐

Yes, in total ………days

Du you regularly use medicines??

No ☐
Yes ☐

I use the following medicines regularly

……………………………………………………………………………………

……………………………………………………………………………………

……………………………………………………………………………………

How satisfied are you with the treatment you have had for depression during the last 3 months?

(1 means you are not at all satisfied, and 10 that you are completely satisfied with the treatment)

1 2 3 4 5 6 7 8 9 10

Thanks for answering the questions!
